# Supplementary material for: Study on the Application of Nitrogen-Doped Holey Graphene in Supercapacitors with Organic Electrolyte
Source: Nanomaterials (Basel). 2023 May 14;13(10):1640. doi: 10.3390/nano13101640 (PMC10222225; doi:10.3390/nano13101640)
Supplement: Supplementary file 1 [file nanomaterials-13-01640-s001.zip › nanomaterials-2335528-supplementary.pdf]

Supplementary Materials

Study on the application of nitrogen-doped holey graphene in supercapacitors with organic electrolyte

Yu-Ren Huang, Nen-Wen Pu*, Guan-Min Wu, Yih-Ming Liu, Ming-Hsien Lin, Yi-Le Kwong, Siou-Cheng Li, Jeng-Kuei Chang, Ming-Der Ger*

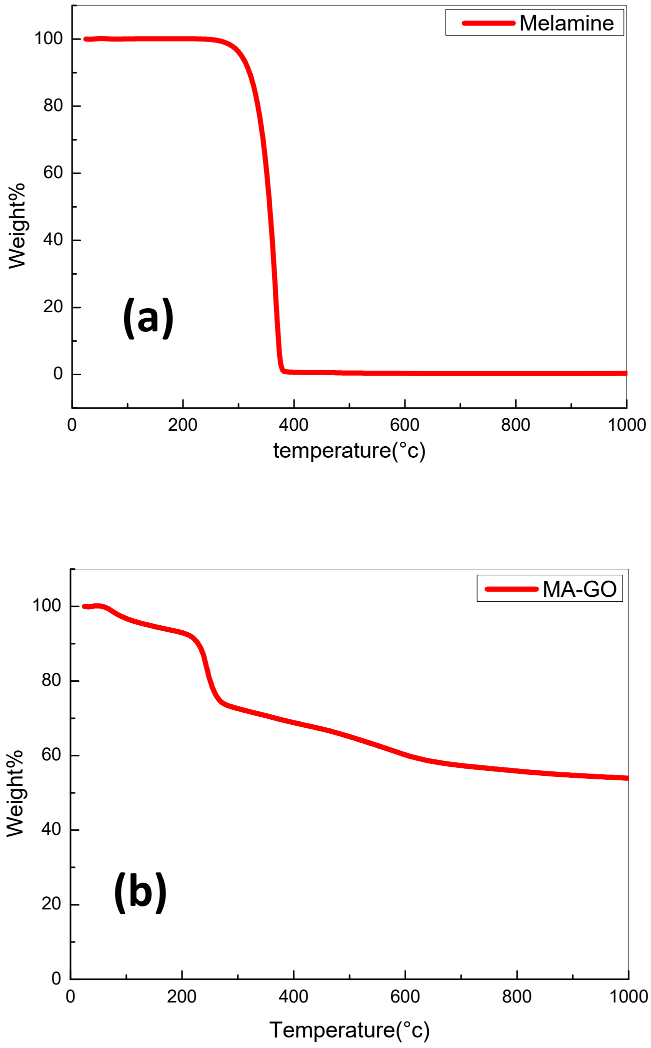

Figure S1. TGA (thermogravimetric analysis) measurement results for (a) melamine and (b) MA-GO. The heating rate was 60°C/min.

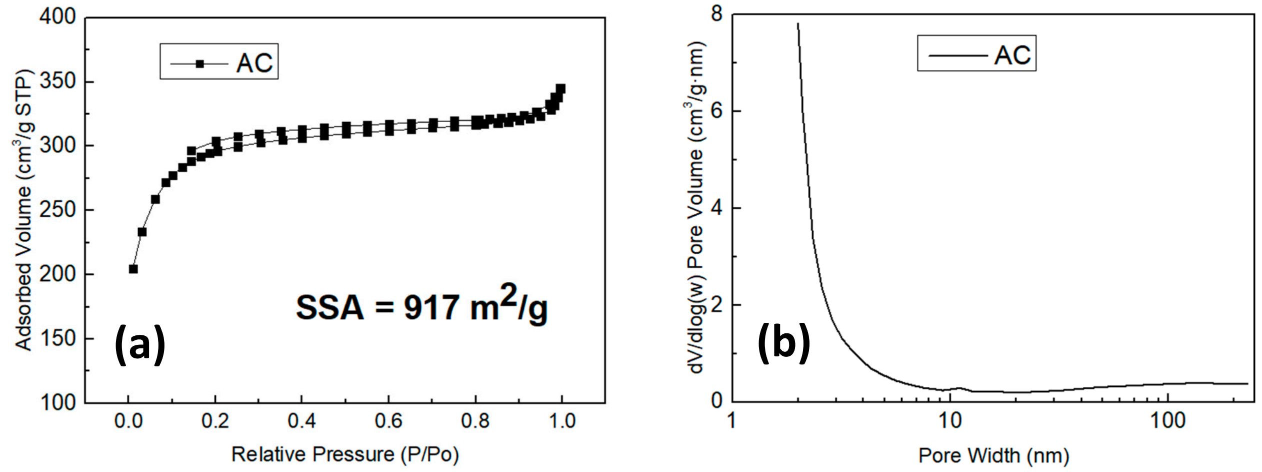

Figure S2. BET analysis for AC: (a) N_2 adsorption/desorption isotherms and (b) pore size distribution.

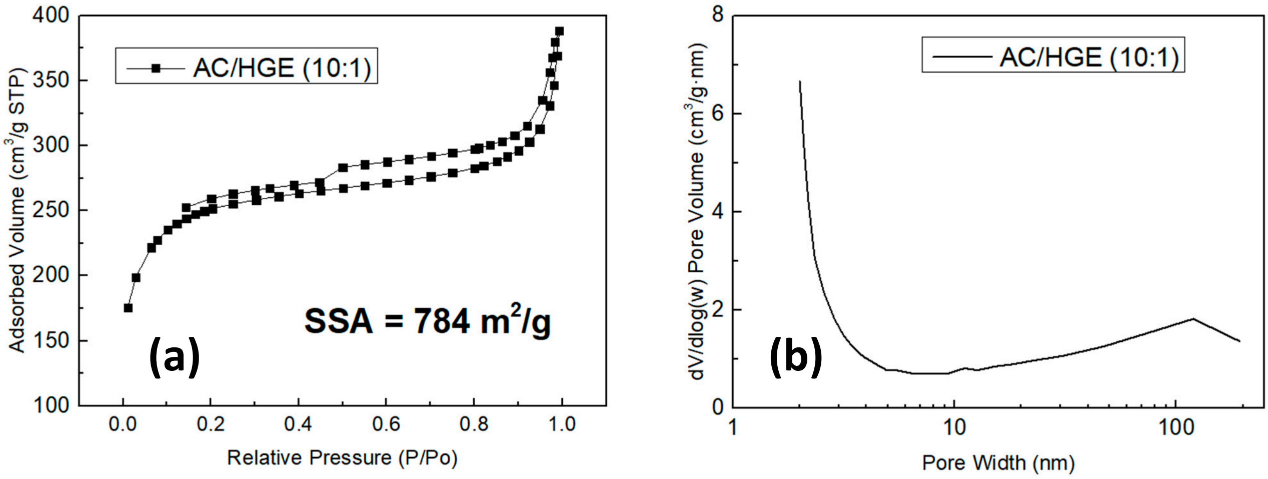

Figure S3. BET analysis for AC/HGE (10:1): (a) N_2 adsorption/desorption isotherms and (b) pore size distribution.

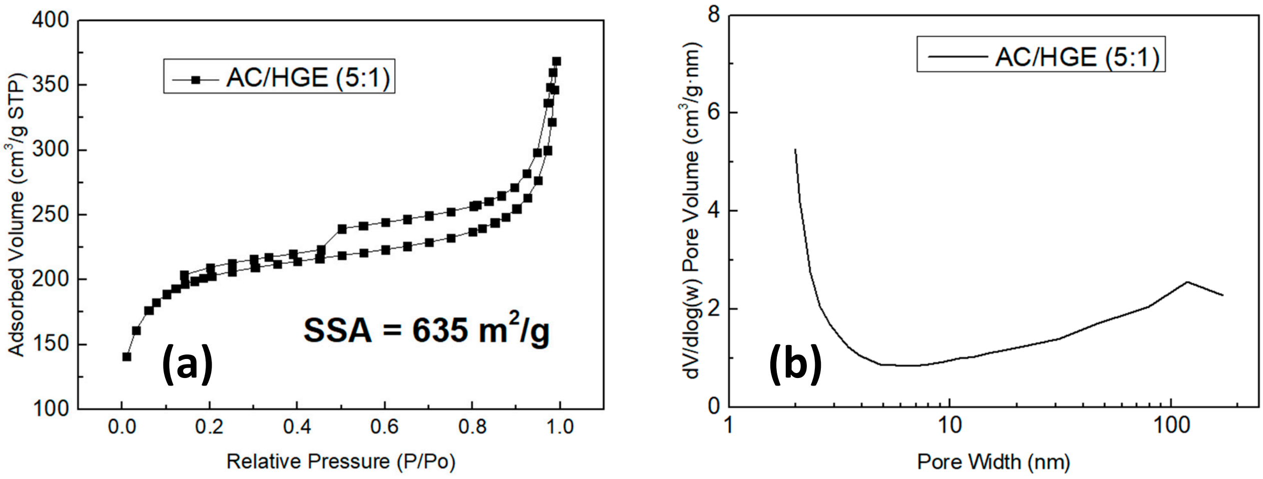

Figure S4. BET analysis for AC/HGE (5:1): (a) N_2 adsorption/desorption isotherms and (b) pore size distribution.

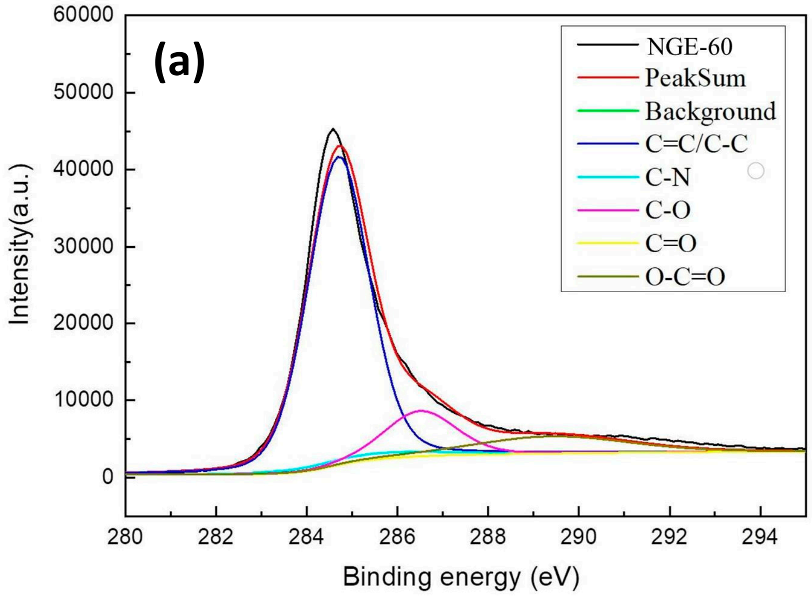

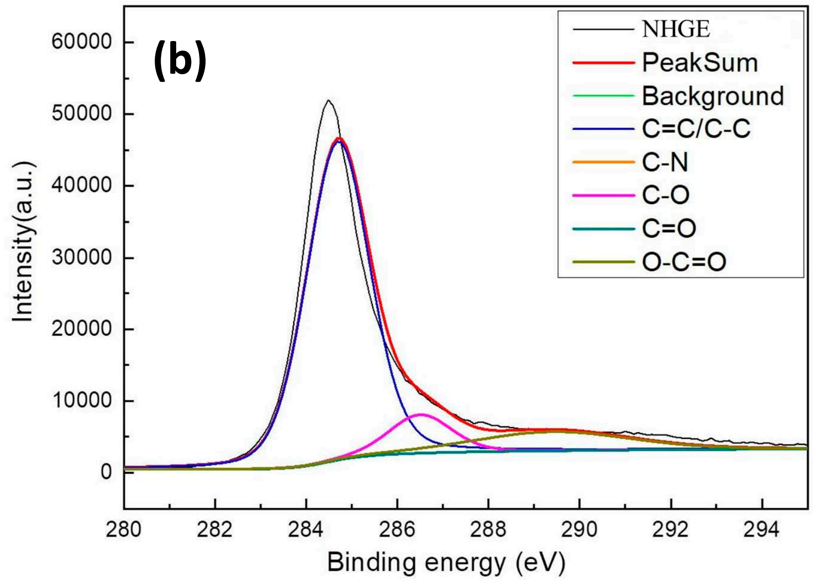

Figure S5. C 1s XPS spectra for (a) N-GE-60 and (b) N-HGE.

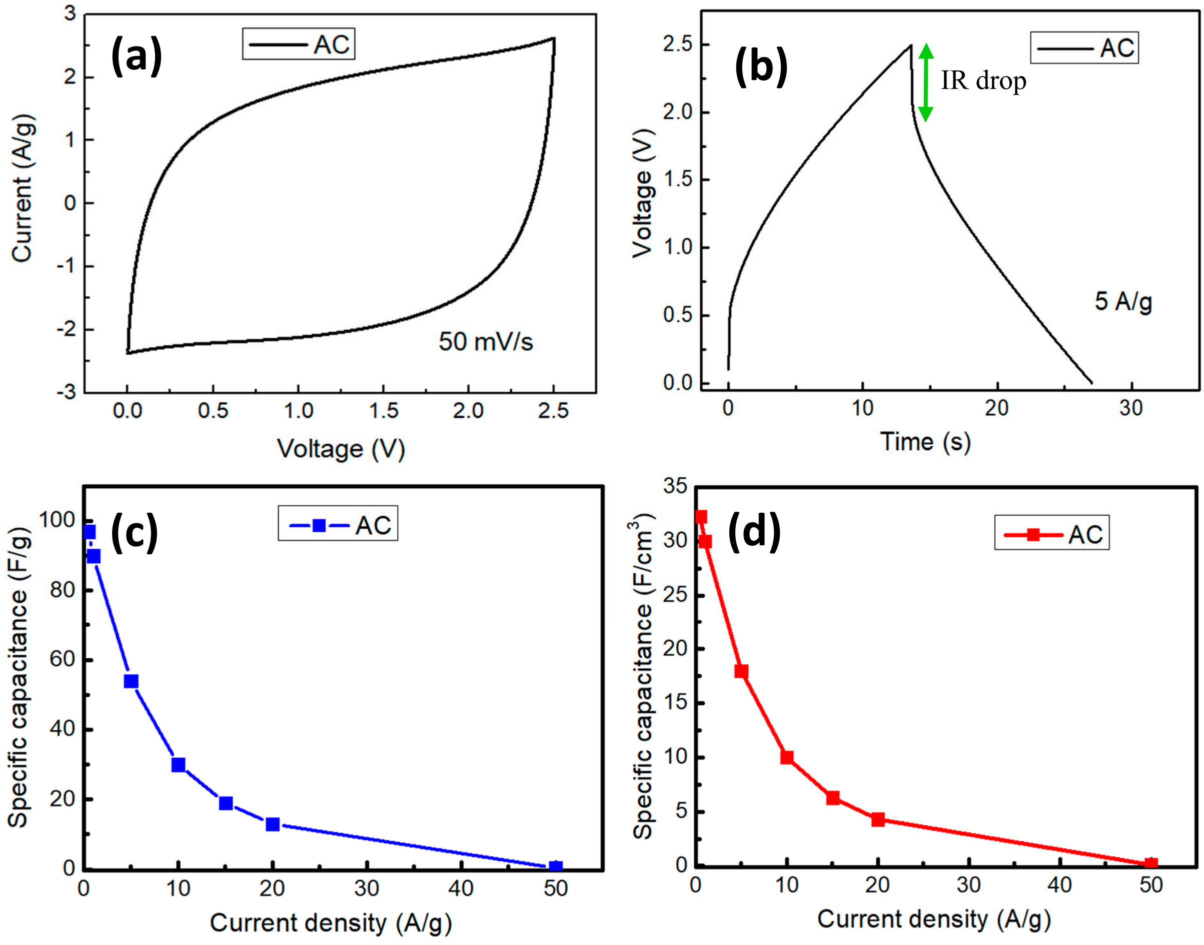

Figure S6. Performances of the SC using AC-only active material: (a) CV curve measured at potential scan rate of 50 mV/s. (b) GCD curve measured at 5 A/g. (c) The gravimetric and (d) volumetric specific capacitances vs. current density.

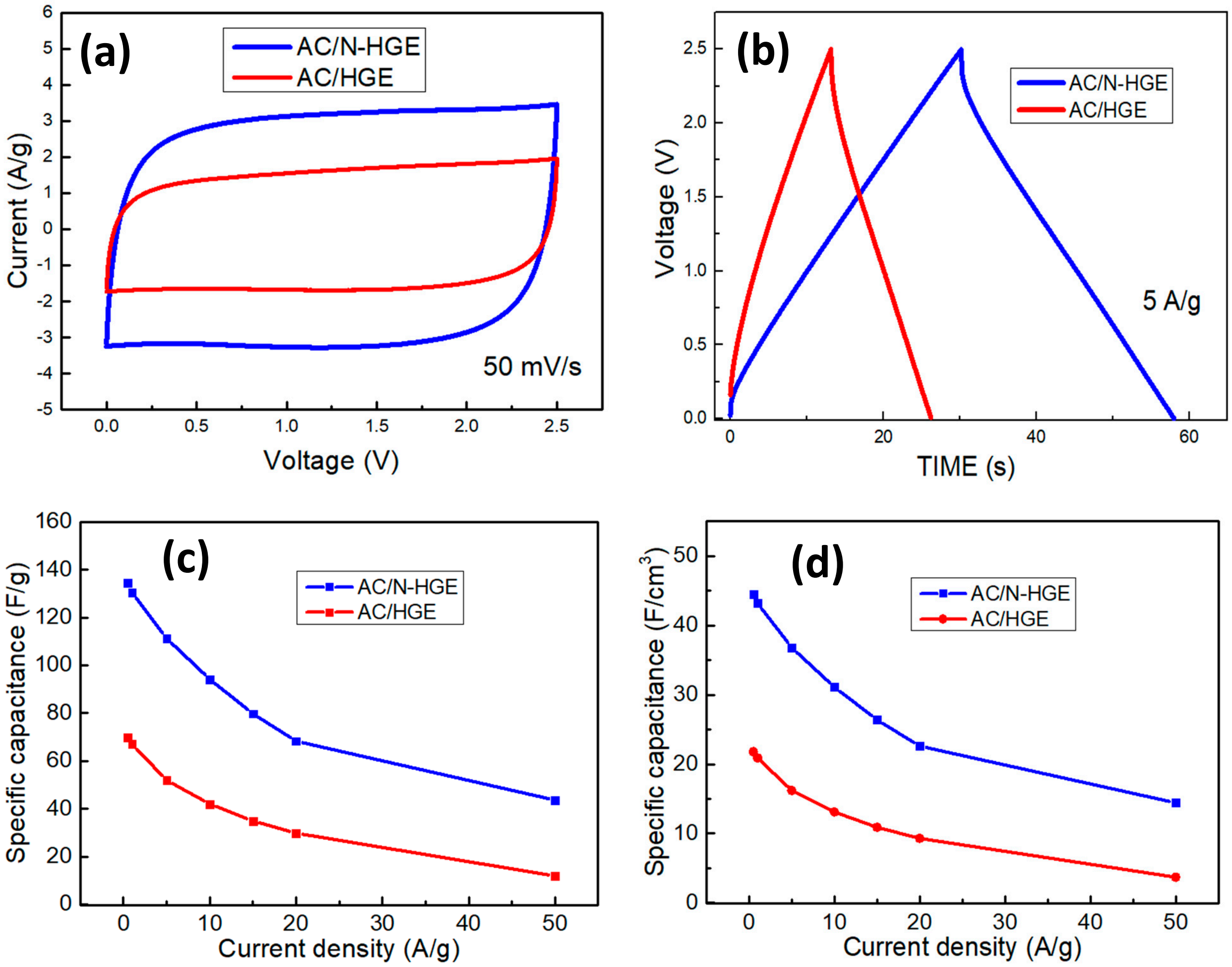

Figure S7. Effects of N-doping on the SC performances revealed by comparing the AC/N-HGE and AC/HGE (without N-doping) electrodes: (a) CV curve measured at potential scan rate of 50 mV/s. (b) GCD curve measured at 5 A/g. (c) The gravimetric and (d) volumetric specific capacitances vs. current density.

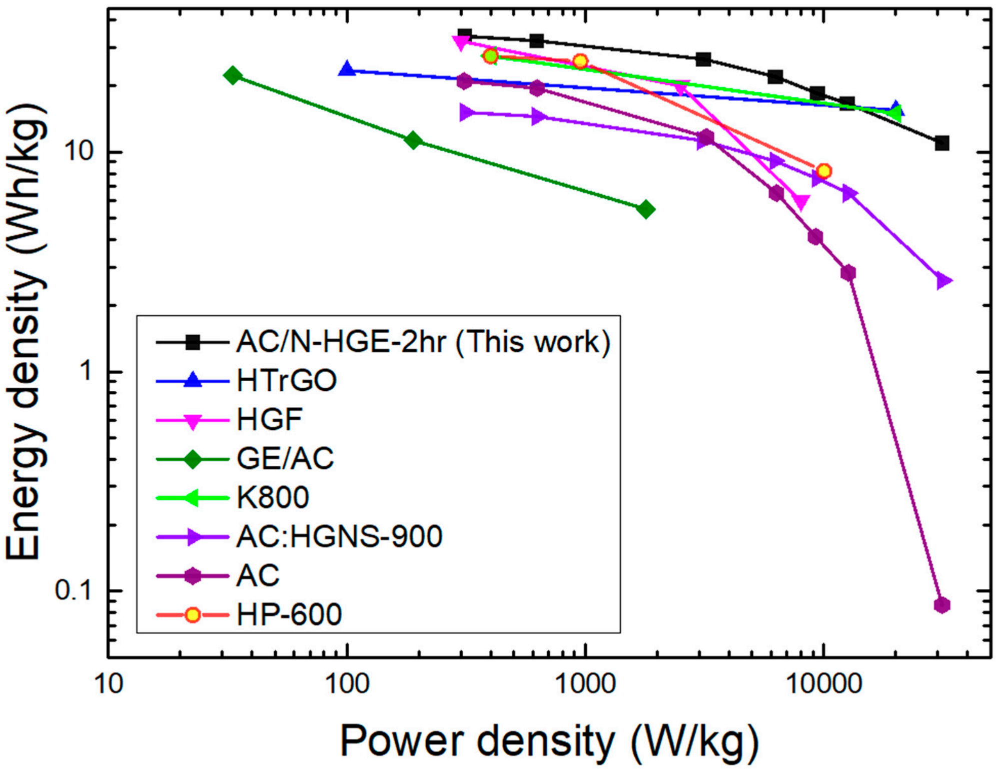

Figure S8. Ragone plot comparing the specific energy vs. specific power performances of our results and other benchmark studies.

Table S1. Details and references for the SCs compared in Figure S8.

Sample	Electrode system	Electrolyte	Potential window	Electrolyte type	Reference
AC/N-HGE-2hr	2	$TEABF_4$/PC	2.5V	Organic	This work
HTrGO	2	KOH	1V	Aqueous	[36]
HGF	2	$EMIMBF_4$/AN	3.5V	Ion liquid	[11]
GE/AC	2	Et_4NBF_4/PC	2.4V	Organic	[37]
K800	2	KOH/PVA	1V	Aqueous	[38]
AC:HGNS-900	2	$TEABF_4$/PC	2.5V	Organic	[18]
AC	2	$TEABF_4$/PC	2.5V	Organic	[18]
HP-600	2	KOH/PVA	1V	Aqueous	[39]
